# Taxogenomic and Physiological Characterization of *Halomonas shizuishanensis* sp. nov. Reveals Osmotic-Adaptation Traits and an Ammonium-Utilization Phenotype

**DOI:** 10.3390/ijms27188335

**Published:** 2026-09-19

**Authors:** Zhen Sun, Beili Sun, Zhichao Deng, Jinchang Han, Yikang Wu, Jinqiu Wang, Pengcheng Gao, Yiming Li, Kai Zhou, Yuxing Wei, Yan Li, Zongli Yao, Qifang Lai

**Affiliations:** 1 East China Sea Fisheries Research Institute, Chinese Academy of Fishery Sciences, 300 Jungong Road, Shanghai 200090, China; sunzhen@ecsf.ac.cn (Z.S.);; 2 School of Environmental Science and Engineering, Shanghai Jiao Tong University, 800 Dongchuan Road, Shanghai 200240, China; 3 Department of Environmental Science and Engineering, University of Shanghai for Science and Technology, Shanghai 200093, China; 4 College of Life Science and Technology, Tarim University, 1487 East Talimu Avenue, Alar 843300, China

**Keywords:** extracellular ammonium decrease, saline–alkaline environment, ectoine, polyphasic taxonomy, *Halomonas shizuishanensis*

## Abstract

Extreme saline–alkaline environments impose simultaneous osmotic and nitrogen stresses, yet the traits that enable microbial persistence and nitrogen utilization under these conditions remain incompletely understood. Here, we isolated and characterized a novel bacterium, *Halomonas shizuishanensis DWK9^T^*, from a saline–alkaline soil (salinity 5.75 g/kg, pH 8.35) in Shizuishan City, Ningxia, China, using a polyphasic taxonomic and genomic approach. Strain DWK9^T^ grew at 0–20% (*w*/*v*) NaCl and pH 7.0–10.0. Although its 16S ribosomal RNA gene shared 98.4% similarity with *H. lutescens Q1UT^T^*, phylogenomic analysis and genome-based indices, including average nucleotide identity (88.12%) and digital DNA-DNA hybridization (32.5%), supported its classification as a distinct species. Genome analysis identified a complete *ectABC* cluster and genes annotated for ammonium assimilation and nitrate/nitrite metabolism, including *glnA*, *gdhA*, *nasA*, *narGK*, and *nirBD*. Physiological experiments showed that extracellular ammonium nitrogen concentrations decreased in inoculated cultures relative to the corresponding sterile control, concomitantly with bacterial growth, while nitrite nitrogen accumulated only to a limited extent. These findings provide genomic and physiological observations relevant to osmotic adaptation and inorganic-nitrogen dynamics in a halotolerant bacterium from a saline–alkaline ecosystem.

## 1. Introduction

Saline–alkaline environments, which are rapidly expanding globally due to climate change and intensive anthropogenic activities, represent one of the most severe frontiers for terrestrial and aquatic ecosystems [1,2]. In these habitats, extreme osmotic stress combined with elevated pH fundamentally disrupts the global biogeochemical nitrogen cycle. While ammonia is rapidly oxidized to nitrate by nitrifying bacteria in typical freshwater systems, elevated salinity and pH in saline–alkaline environments severely inhibit conventional nitrifying and denitrifying communities, particularly nitrite-oxidizing bacteria [3]. Consequently, closed saline–alkaline aquatic systems frequently experience ammonia accumulation levels exceeding 5–10 mg/L [4]. More dangerously, the high pH shifts the chemical equilibrium toward un-ionized ammonia (NH_3_), which is highly toxic to aquatic life. Furthermore, the disrupted nitrogen cycle often stalls at the intermediate step, leading to massive accumulations of toxic nitrite (NO_2_^−^). Resolving this dual challenge, surviving extreme osmotic pressure while mitigating toxic nitrogen accumulation, is not merely an ecological niche problem, but a pressing global imperative for ecosystem restoration, agricultural sustainability, and the safe expansion of aquaculture.

The genus *Halomonas* comprises highly adaptable microbial players that have colonized diverse extreme hypersaline habitats [5,6,7,8]. While their robust halotolerance and baseline biogeochemical capabilities are well-documented, a critical knowledge gap remains regarding their eco-evolutionary strategies under severe nitrogen-polluted saline–alkaline conditions. How do native microbes genetically and metabolically rewire their networks to balance the high energy demands of osmotic survival with the need to detoxify hyper-accumulated nitrogen? Current research often focuses on nitrification and denitrification pathways [5]. While certain *Halomonas* species possess strong salt tolerance and excellent ammonia assimilation capabilities, they are typically adapted to carbon-intensive and energy-rich eutrophic wastewater [9]. In contrast, ammonium assimilation under carbon-limited saline–alkaline conditions require efficient intracellular nitrogen incorporation strategies. In many bacteria, this process is primarily mediated by the glutamine synthetase/glutamate synthase (GS/GOGAT) pathway, where *glnA*-encoded glutamine synthetase catalyzes the ATP-dependent incorporation of ammonium into glutamine and contributes to cellular nitrogen homeostasis [10]. However, whether halotolerant *Halomonas* species employ similar regulatory strategies or possess specialized genomic adaptations to couple ammonium assimilation with osmotic stress resistance remains largely unexplored.

In this study, we isolated strain DWK9^T^ from saline–alkaline soil and determined its taxonomic position using a polyphasic approach integrating phenotypic, chemotaxonomic, phylogenetic, and phylogenomic analyses. In parallel, we investigated genome-encoded features associated with osmotic adaptation and nitrogen metabolism and characterized bacterial growth together with extracellular ammonium nitrogen (NH_4_^+^-N), nitrite nitrogen (NO_2_^−^-N), and nitrate nitrogen (NO_3_^−^-N) dynamics during cultivation. These integrated analyses establish the taxonomic identity of strain DWK9^T^ while providing insights into its physiological adaptation and nitrogen-utilization characteristics under saline–alkaline conditions.

## 2. Results and Discussion

### 2.1. A Distinct Phenotypic and Chemotaxonomic Fingerprint

**Figure 1:**
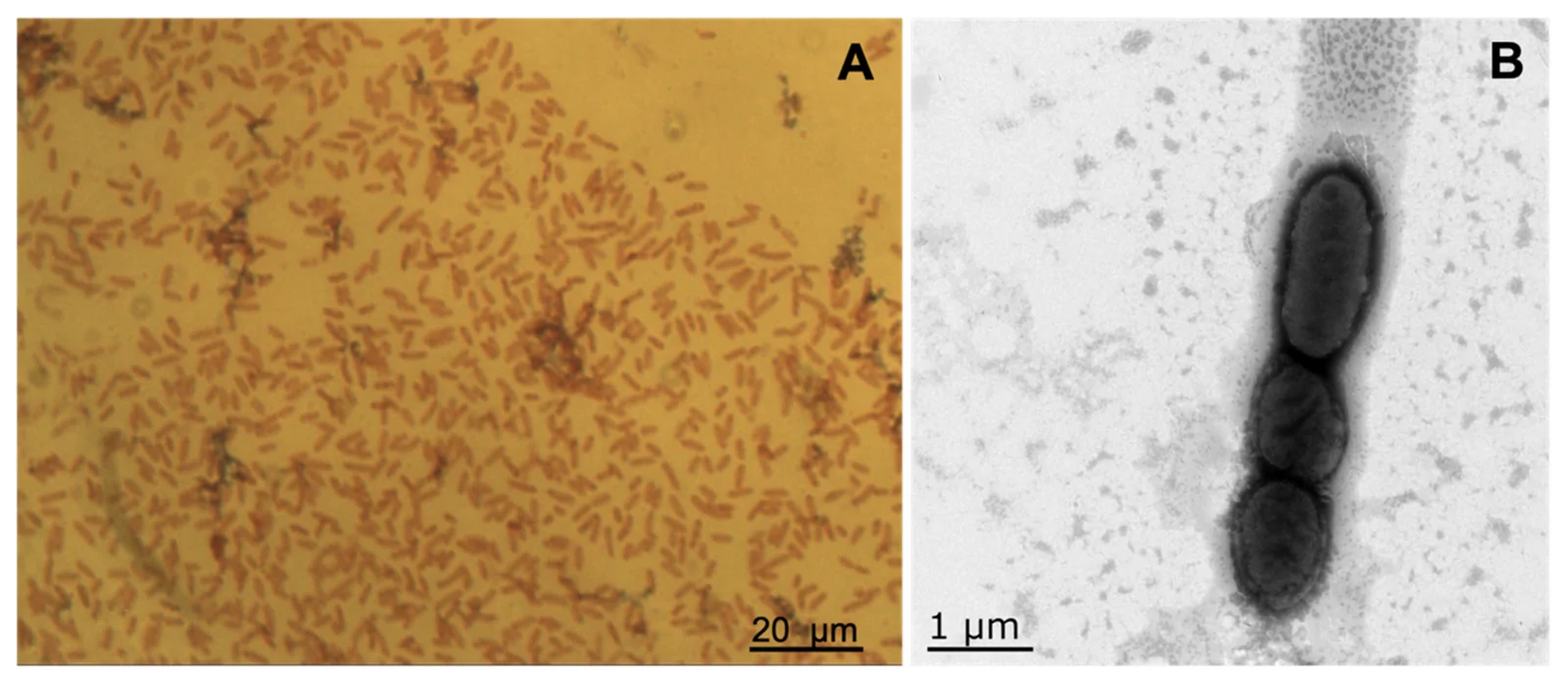
Morphological characteristics of *H. shizuishanensis *DWK9^T^. (**A**) Gram stain under 100× oil lens, and (**B**) whole-cell transmission electron micrograph of cells grown in LB broth medium.

Strain DWK9^T^ exhibits a combination of phenotypic and chemotaxonomic traits that, while consistent with the genus *Halomonas*, immediately distinguished it as a unique member. On Luria-Bertani (LB) agar, it formed smooth, moist, yellowish-white colonies with entire margins. It is a Gram-negative, aerobic, motile rod (0.5–0.8 µm × 1.2–2.0 µm) with a single polar flagellum (Figure 1). Its growth profile reflects a clear adaptation to its native environment, showing pronounced halotolerance (growth at 0–20% *w*/*v* NaCl, optimum 9%) and alkaliphily (growth at pH 7.0–10.0, optimum 8.0–9.0), with a temperature optimum of 28 °C.

The uniqueness of strain DWK9^T^ is clearly defined by its highly specialized biochemical fingerprint (Table 1). While it shares core aerobic traits like catalase and nitrate reduction positivity with the genus, it exhibits a restricted carbon-utilization profile and lacks oxidase activity—a specific phenotypic signature that sharply differentiates it from its closest relative, *H. lutescens *Q1UT^T^ [11]. It does not hydrolyze gelatin, starch, aesculin, or urea and is negative for both the Methyl Red and Voges–Proskauer tests. Strain DWK9^T^ is oxidase-negative, a stark contrast to the positive result in *H. lutescens *Q1UT^T^. This separation is further solidified by its carbon metabolism; unlike *H. lutescens *Q1UT^T^, strain DWK9^T^ cannot utilize D-glucose or D-cellobiose for growth. Furthermore, its positive indole production distinguishes it from other relatives like *H. zhaodongensis *NEAU-ST10-25T^T^ [12]. This unique combination of diagnostic traits, particularly its negative oxidase activity and specific carbon utilization, paints the picture of a specialist adapted to the fluctuating redox and nutrient conditions of saline–alkaline soils.

This distinction is powerfully reinforced by its chemotaxonomic profile. The major fatty acids were C18:1ω7c (53.05%), C16:0 (19.64%), and C16:1ω7c (17.25%), a composition consistent with its placement in the genus *Halomonas* (Table 2). The quantitative profile provides clear and compelling diagnostic markers. Notably, the proportion of C18:1ω7c (53.05%) in strain DWK9^T^ was intermediate, positioned below that of key relatives like *H. lutescens *Q1UT^T^ (58.6%) and *H. zhaodongensis *NEAU-ST10-25T^T^ (62.3%), yet distinctly higher than that of *H. songnenensis *NEAU-ST10-39^T^ (47.2%) [14]. More decisively, the abundance of C16:0 (19.64%) was markedly higher than in all compared type strains, which ranged from only 10.1% to 17.6%. Furthermore, the significant presence of C17:0 cyclo (3.26%), a fatty acid absent or found only in trace amounts in several of its close relatives, constitutes another key differentiator. This unique quantitative signature—defined by an elevated proportion of C16:0, an intermediate level of C18:1ω7c, and the notable presence of C17:0 cyclo—constitutes a robust chemotaxonomic fingerprint. This structural pre-adaptation provides the first line of defense against the osmotic shock characteristic of its saline–alkaline niche, setting the stage for its unique intracellular metabolic strategies.

### 2.2. Genomic Analysis Defines a Novel Species

The initial phylogenetic investigation was based on the near-complete 16S rRNA gene sequence (1422 bp). Comparative analysis revealed that strain DWK9^T^ shares the highest sequence similarity of 98.4% with *Halomonas lutescens *Q1UT^T^ [11], followed by close relationships to *H. zhaodongensis *NEAU-ST10-25T^T^ (97.7%) [12], *H. hydrothermalis *Slthf2^T^ (97.6%) [15], and *H. lionensis *RHS90^T^ (97.6%) [13] (Figure S1). Despite this high similarity, phylogenetic reconstructions using neighbor-joining (NJ), maximum-likelihood (ML), and maximum-parsimony (MP) methods consistently placed strain DWK9^T^ on a distinct, well-supported monophyletic branch (e.g., 95% bootstrap support in the NJ tree; Figures S1–S3). However, because species within the genus *Halomonas* frequently exhibit 16S rRNA gene similarities exceeding the traditional 97% threshold, Whole-genome sequencing is imperative to provide higher taxonomic resolution.

**Figure 2:**
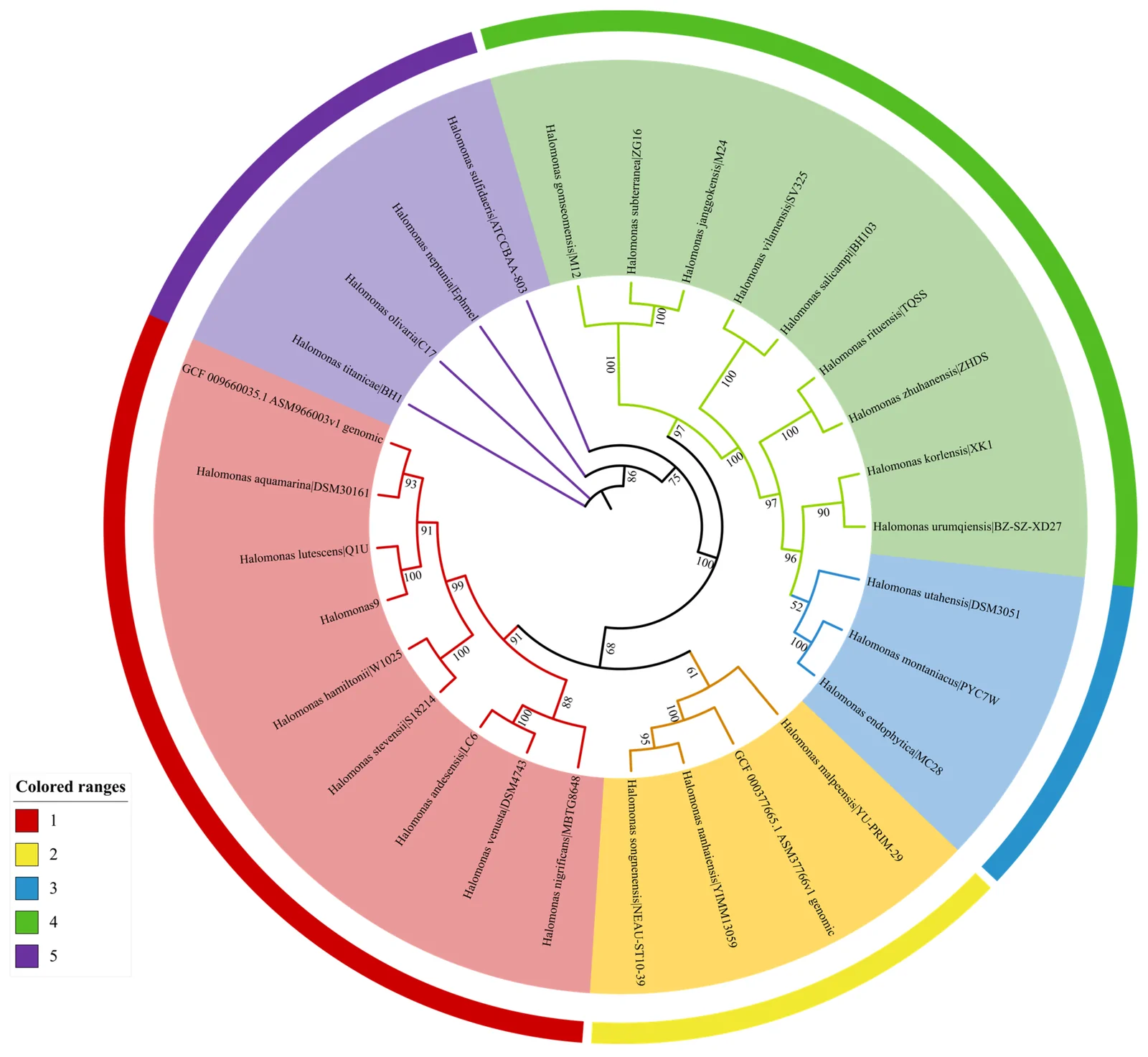
Maximum-likelihood phylogenomic tree based on 120 concatenated bacterial single-copy marker genes showing the evolutionary position of *H. shizuishanensis *DWK9^T^ among closely related species within the genus *Halomonas*. The marker genes were extracted and aligned using GTDB-Tk, and the tree was inferred using IQ-TREE 2. Numbers at the nodes indicate Ultrafast Bootstrap support values (based on 1000 replicates; only values > 60% are shown).

To conclusively determine its taxonomic status, whole-genome sequencing was conducted. The DNA guanine-plus-cytosine (G+C) content of strain DWK9^T^ was determined to be 55.9%, which is markedly lower than that of its closest relative, *H. lutescens *Q1UT^T^ (61.5%) (Table 3), providing an initial layer of evidence for genomic divergence. Furthermore, the reliance on single-gene phylogeny was entirely superseded by our phylogenomic analysis. The highly robust phylogenomic tree, inferred from 120 concatenated ubiquitous bacterial single-copy core genes (Figure 2), unequivocally resolved DWK9^T^ into a distinct, well-separated clade within the genus *Halomonas* with high bootstrap support (99.7%). This unique phylogenetic placement was further corroborated by an auxiliary tree based on 31 essential housekeeping genes (Figure S4). Such topological consistency across different multilocus datasets clearly demarcates the strain as an independent evolutionary lineage.

Ultimately, the gold-standard Overall Genome Relatedness Indices (OGRI) delivered the final verdict for species delineation. The average nucleotide identity (ANI) value between strain DWK9^T^ and *H. lutescens *Q1UT^T^ was only 88.12% (Table 3, Figure S5), which falls dramatically below the established 95–96% species boundary [17]. Furthermore, the digital DNA-DNA hybridization (dDDH) value against its closest relative was merely 32.5%, well below the universally accepted 70% threshold [18]. The ANI values against other reference *Halomonas* species were even lower (ranging from 83.35% to 86.70%), confirming its profound genomic isolation.

Although polar lipid and respiratory quinone profiles were not determined, the combined phenotypic, phylogenomic, ANI, and dDDH evidence supports the taxonomic placement of the strain. More importantly, in the era of taxogenomics, the congruence of disparate G+C content, a multi-locus supported phylogenomic lineage, and exceptionally low ANI/dDDH values provides absolute resolution. This multi-layered genomic evidence definitively anchors the status of strain DWK9^T^ as a novel *Halomonas* species, superseding the necessity for traditional chemotaxonomic markers. Having unequivocally established its taxonomic novelty, we subsequently interrogated its genome to uncover the functional adaptations that allowed this distinct evolutionary lineage to colonize such a harsh environment. The formal species description and diagnostic characteristics of *Halomonas shizuishanensis* sp. nov. are summarized in Table 4.

### 2.3. Genomic Blueprint for Adaptation to a Saline–Alkaline Niche

**Figure 3:**
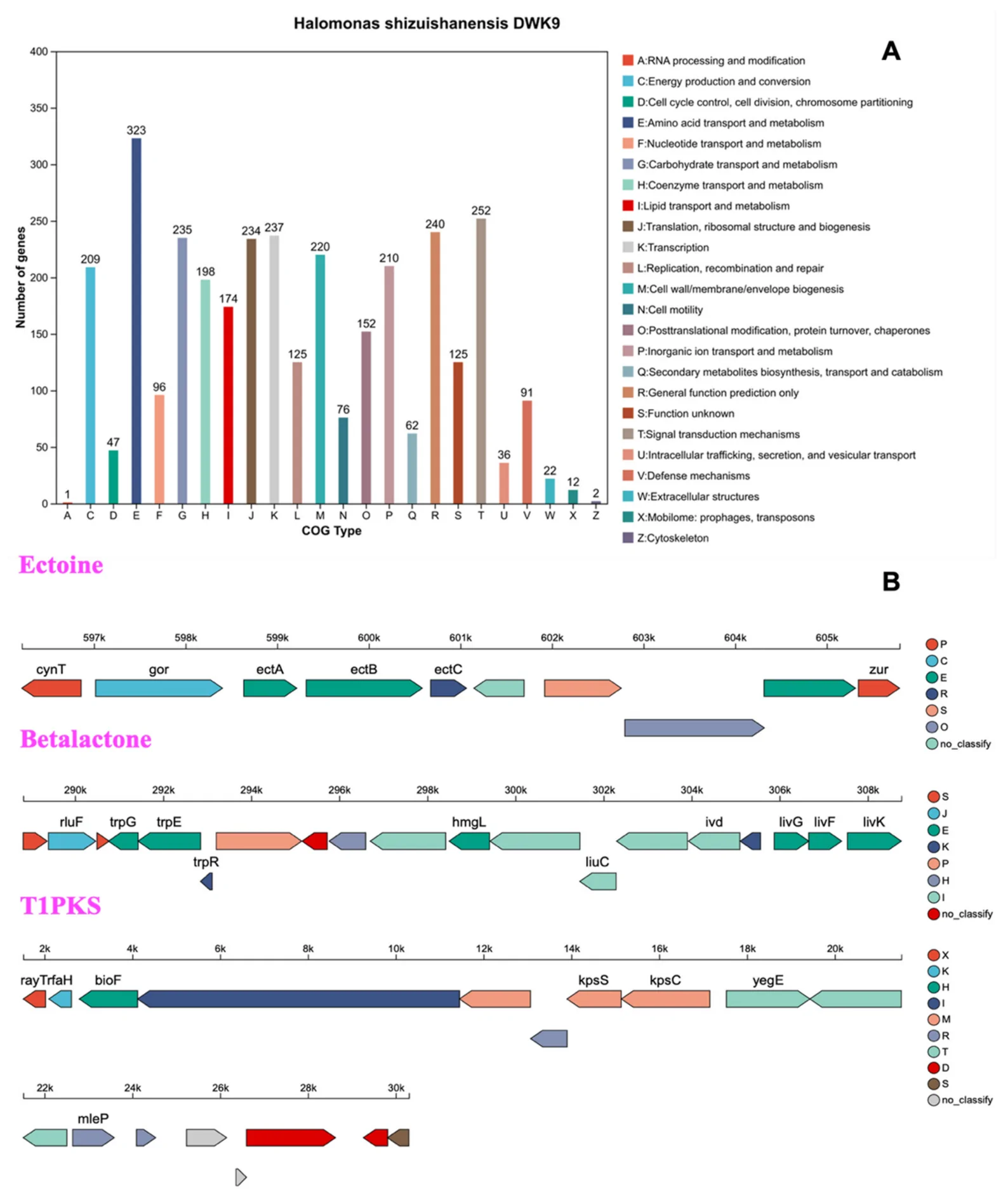
Clusters of Orthologous Groups of proteins (COG) functional classification and secondary metabolite biosynthetic gene clusters of the *H. shizuishanensis *DWK9^T^. (**A**) Distribution of genes across COG functional categories. The bar chart shows the number of genes assigned to each COG class, with detailed category descriptions provided in the legend. (**B**) Predicted biosynthetic gene clusters for ectoine, betalactone, and T1PKS identified by antiSMASH v6.0. ORFs are represented by arrows, color-coded by predicted function based on Blastp alignments. Gene cluster types and genomic locations are indicated above each cluster.

The 3.81 Mb genome of strain DWK9^T^ contains 3468 predicted genes (Table S1). Functional annotation assigned 8.23% and 6.74% of genes to signal-transduction and transcription categories, respectively (Figure 3A, Table S2), whereas 5.78% and 3.90% were assigned to inorganic-ion transport and metabolism, and lipid transport and metabolism, respectively. The occurrence of genes in these functional categories suggests that strain DWK9^T^ possesses genetic features potentially relevant to environmental sensing, ion homeostasis, and membrane-associated functions.

The genome of strain DWK9^T^ harbors a complete ectoine-biosynthesis gene cluster (*ectABC*) (Figure 3B), suggesting the genomic potential for ectoine biosynthesis. As a well-characterized osmoprotectant [19], ectoine may contribute to the tolerance of strain DWK9^T^ to osmotic stress. The genome also contains genes associated with ammonium assimilation, including *glnA* and *gdhA* for ammonium assimilation, *nasA* for assimilatory nitrate reduction, and *narGK* and *nirBD* for nitrate/nitrite reduction. Together, these annotations indicate the potential capacities for compatible-solute biosynthesis and diverse nitrogen-related metabolic processes, providing a genomic framework for interpreting the physiological characteristics of strain DWK9^T^.

**Figure 4:**
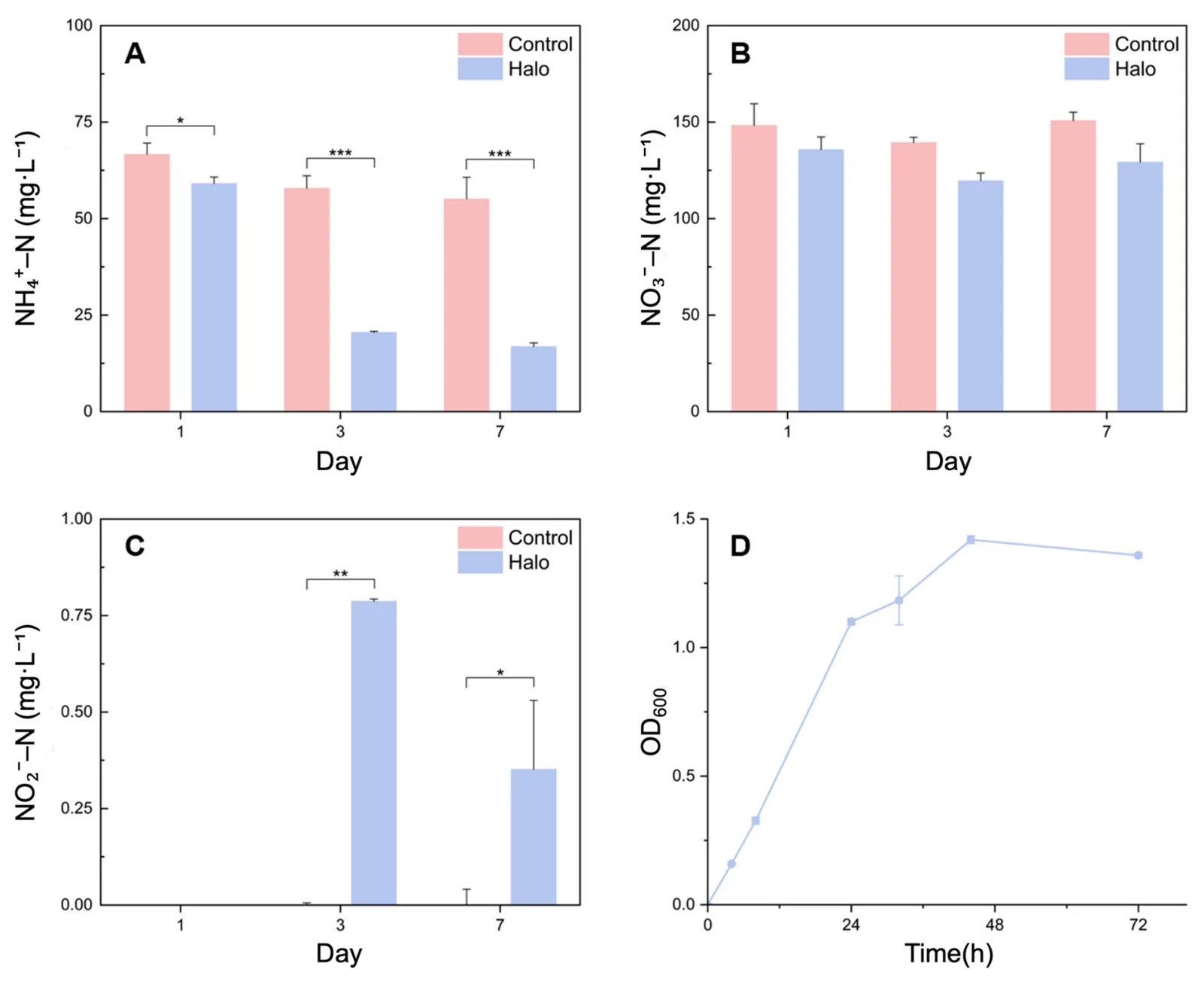
Growth and extracellular inorganic nitrogen profiles of Halomonas shizuishanensis DWK9^T^ during cultivation. (**A**) NH_4_^+^-N, (**B**) NO_3_^−^-N, and (**C**) NO_2_^−^-N were measured on days 1, 3, and 7; (**D**) growth was monitored as OD_600_ during the first 72 h. Data are presented as mean ± SD from three independent biological replicate cultures per treatment, measured repeatedly over time. For each nitrogen species, treatment, time, and treatment × time interaction was analyzed using two-factor repeated-measures ANOVA. NO_2_^−^-N was analyzed after signed square-root transformation because several values were close to the analytical zero. Brackets in panels A and C indicate Holm-adjusted treatment comparisons within each sampling time: * *p* < 0.05; ** *p* < 0.01; *** *p* < 0.001. For NO_3_^−^-N, the treatment × time interaction was not significant.

The optical density at 600 nm (OD_600_) of strain DWK9^T^ increased rapidly during the first 24 h, reached a maximum at approximately 48 h, and remained high at 72 h (Figure 4D). In parallel, extracellular NH_4_^+^-N in the inoculated cultures decreased from approximately 59 mg N/L on day 1 to approximately 20 mg N/L on day 3 and remained at a similarly low level on day 7 (Figure 4A). A two-factor repeated-measures ANOVA identified significant effects of treatment, time, and treatment × time interaction on extracellular NH_4_^+^-N concentrations (all *p* < 0.001). Holm-adjusted pairwise comparisons showed that NH_4_^+^-N concentrations were lower in inoculated cultures than in the corresponding sterile controls on days 1, 3, and 7 (*p* = 0.018, *p* < 0.001, and *p* < 0.001, respectively; Figure 4A). NO_2_^−^-N showed a transient, low-level increase, reaching approximately 0.79 mg N/L on day 3 before declining thereafter (Figure 4C). For NO_3_^−^-N, treatment and time effects were significant, whereas the treatment × time interaction was not significant (Figure 4B). Overall, these results demonstrate a pronounced culture-associated decrease in extracellular NH_4_^+^-N between days 1 and 3, followed by the maintenance of relatively low concentrations through day 7.

### 2.4. Integrated Genomic and Physiological Features Associated with Saline–Alkaline Adaptation and Extracellular Nitrogen Dynamics

The phenotypic and genomic data together characterize strain DWK9^T^ as a halotolerant and alkalitolerant member of the genus *Halomonas*. Its broad salt and pH growth ranges are accompanied by a complete *ectABC* cluster, which provides a genetic basis for the potential synthesis of ectoine, a well-established compatible solute. In addition, the relatively high proportion of C16:0 and the presence of C17:0 cyclo distinguish DWK9^T^ from related type strains at the chemotaxonomic level. Considered together, these features are consistent with the capacity of the strain to persist under saline–alkaline cultivation conditions.

The nitrogen profiles described in Section 2.3 provide physiological context for the nitrogen-metabolism genes identified in the genome. Compared with the corresponding sterile controls, inoculated cultures showed a greater decrease in extracellular NH_4_^+^-N during the period of increasing OD_600_, whereas NO_2_^−^-N accumulated only transiently. For NO_3_^−^-N, the absence of a significant treatment × time interaction indicates that the treatment-associated difference remained relatively consistent across the sampling period. These extracellular nitrogen profiles, however, do not resolve the intracellular fate of ammonium or demonstrate the activity of specific annotated pathways. Rather, the genomic data identify *glnA*, *gdhA*, *nasA*, *narGK*, and *nirBD* as candidate genes potentially associated with the observed nitrogen dynamics. Supporting their environmental responsiveness, previous transcriptomic and RT-qPCR analyses of the same strain under alkali–photocatalytic stimulation revealed condition-dependent changes in the expression of these genes [7]. Together, these findings provide a basis for future studies directly linking specific nitrogen-metabolism pathways to the observed physiological responses.

The nitrogen-related features of DWK9^T^ can also be considered in the context of those reported for other halotolerant *Halomonas* strains. Nitrogen removal by *H. salifodinae *Y5 has been attributed predominantly to simultaneous nitrification and denitrification [20], whereas *Halomonas* sp. W07 harbors genetic features associated with dissimilatory nitrate reduction to ammonium and ammonium assimilation [9]. Under the cultivation conditions used in the present study, DWK9^T^ was characterized by a culture-associated decrease in extracellular NH_4_^+^-N with only limited accumulation of NO_2_^−^-N. At the genomic level, the co-occurrence of *glnA/gdhA* with *narGK* and *nirBD* in DWK9^T^ shows a pattern comparable to that reported for *Halomonas* sp. W07. Because these three strains were investigated using different media, cultivation conditions, and analytical endpoints, these comparisons are intended to place the nitrogen-related characteristics of DWK9^T^ within the broader physiological and genomic diversity of *Halomonas*, rather than to directly compare their nitrogen-removal efficiencies.

**Figure 5:**
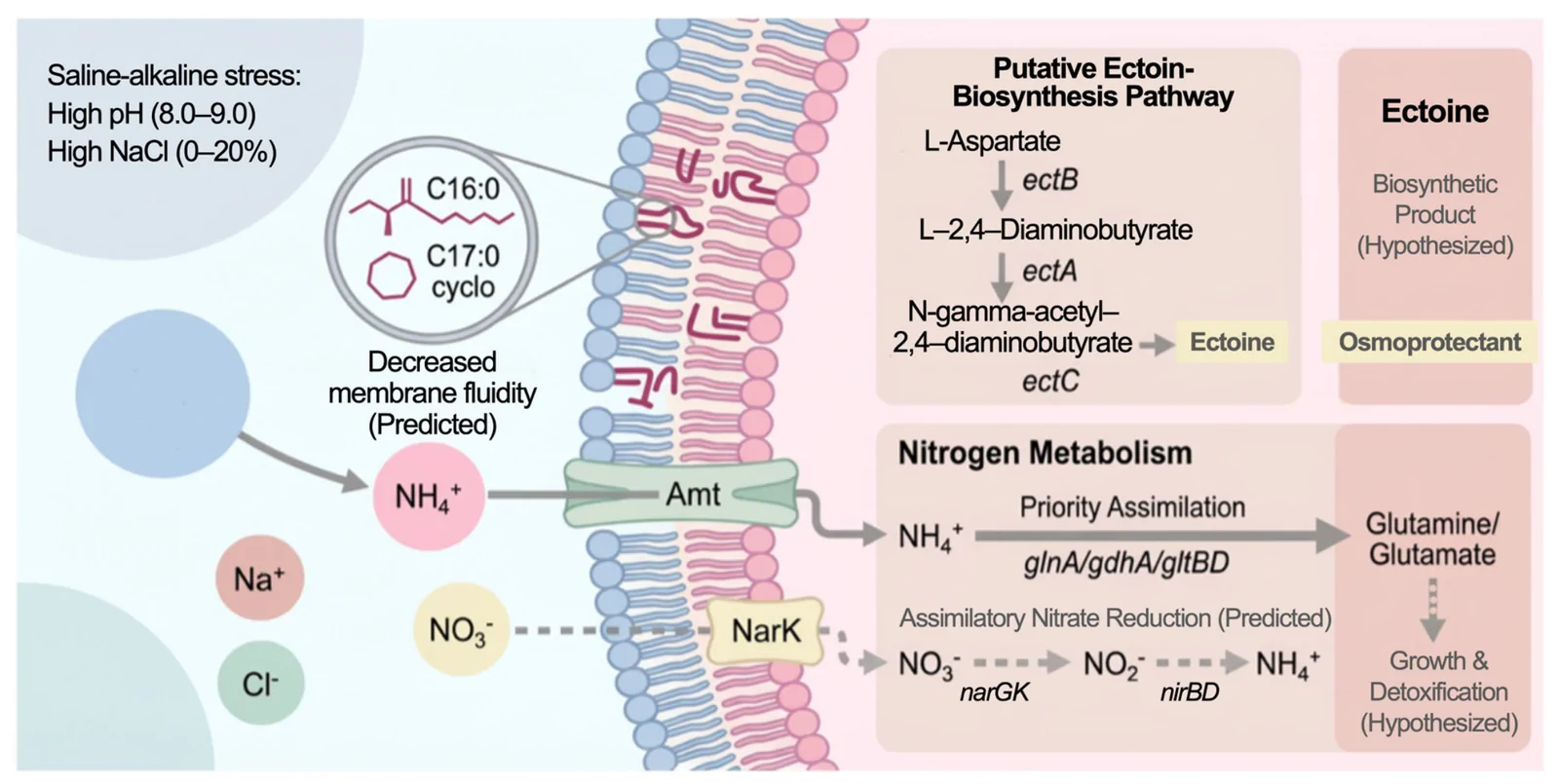
Conceptual framework integrating the observed extracellular nitrogen profiles with genome annotations related to osmotic adaptation and nitrogen metabolism in *H. shizuishanensis *DWK9^T^. The scheme presents genomic annotations and proposed functional relationships in the context of the culture-level observations. Solid arrows: genomic and transcriptomic support; dashed arrows: genomic support only.

Taken together, the growth characteristics, *ectABC* cluster, nitrogen-related gene repertoire, and extracellular nitrogen profiles provide an integrated view of strain DWK9^T^ (Figure 5). This conceptual framework links the observed culture-level responses with candidate functions inferred from genome annotation, while avoiding assumptions about pathway activity that were not directly tested in the present study. The broad salt and pH growth ranges of DWK9^T^, together with its reproducible culture-associated nitrogen profiles, provide a basis for further evaluation under defined saline–alkaline conditions. Future studies incorporating nitrogen mass-balance analysis, quantification of intracellular nitrogen incorporation and gaseous nitrogen products, and direct assessment of pathway activity will be needed to resolve nitrogen fate and establish the mechanisms underlying the observed physiological responses. Such evidence will also be essential for determining whether these characteristics can be translated into practical applications for nitrogen management in saline–alkaline environments.

## 3. Materials and Methods

### 3.1. Strain Isolation, Cultivation, and Deposition

Strain DWK9^T^ was isolated from the surface layer (0–20 cm depth) of waterlogged saline–alkaline soil collected in Shizuishan, China (38°53′52.638″ N, 106°19′39.222″ E). The sampling site had a soil pH of 8.35, a salinity of 5.75 g/kg, an organic matter content of 11 g/kg, and a microaggregate proportion exceeding 60%. These physicochemical characteristics provide the environmental context for the isolation of strain DWK9^T^.

Soil samples were processed within 24 h of collection. Serially diluted soil suspensions were inoculated into a denitrifying medium (DM) [21] containing, per liter, 4.7 g C_4_H_4_Na_2_O_4_·6H_2_O, 1.0 g KNO_3_, 7.9 g Na_2_HPO_4_·7H_2_O, 0.3 g NH_4_Cl, 0.1 g MgSO_4_·7H_2_O, pH 8.2, and 2 mL trace element solution [22]. After incubation for 7 d at 28 °C with shaking at 180 rpm, enrichment cultures were transferred to bromothymol blue (BTB) agar plates [23] for colony isolation. Strain DWK9^T^ was purified by repeated streaking on LB agar.

In parallel with the enrichment-based isolation procedure, serial dilutions of soil suspensions from multiple sampling batches were directly spread onto LB agar plates. Isolates with 16S rRNA gene sequences identical to that of strain DWK9^T^ were recovered from each sampling batch, demonstrating the repeated recovery of DWK9^T^-like isolates from the sampled soil under the LB cultivation conditions used. Media were prepared in-house according to the cited published protocols, and all constituent reagents were of analytical grade, sourced from Sinopharm Chemical Reagent Co., Ltd., (Shanghai, China) and Sangon Biotech (Shanghai) Co., Ltd. (Shanghai, China). The type strain DWK9^T^ has been deposited in the China General Microbiology Culture Collection Center (CGMCC 27980^T^) and the Korean Collection for Type Cultures (KCTC 18764^T^).

### 3.2. Phenotypic, Microscopic, and Growth Conditions

A comprehensive phenotypic characterization was conducted to define the morphological, physiological, and biochemical profile of strain DWK9^T^. Cell morphology was initially examined using cells from a 48 h culture on LB agar, and Gram staining was observed under a fluorescence inverted microscope (IX 71, Olympus Corporation, Tokyo, Japan). For transmission electron microscopy (TEM), the bacterial suspension was placed on a carbon-coated copper grid and allowed to dry. The whole-cell micrographs were captured using a Tecnai 12 TEM (Philips, Eindhoven, The Netherlands) operating at an accelerating voltage of 120 kV (Figure 1) [24]. To establish its fundamental growth boundaries, the strain’s response to key environmental variables was systematically quantified. The temperature range for growth was tested in LB broth at incubations of 4, 10, 15, 20, 25, 28, 30, 35, 40, and 45 °C. Salt tolerance was assessed in LB broth supplemented with NaCl at concentrations of 0, 1, 3, 5, 7, 9, 11, 14, 20, and 25% (*w/v*). The pH range was determined in buffered LB broth, using 0.1 M citrate/0.2 M phosphate buffer for pH 5.0–6.0, 0.05 M Tris/ HCl buffer for pH 7.0–9.0, and 0.1 M Na_2_CO_3_/NaHCO_3_ buffer for pH 10.0. In all cases, growth was monitored by measuring OD_600_ using a microplate reader (Epoch, BioTek instruments, Inc., Winooshi, VT, USA).

The strain’s metabolic repertoire was further detailed through a suite of standard biochemical assays as described by Smibert and Krieg [25], including tests for catalase and oxidase activities, hydrogen sulfide production, nitrate reduction, Methyl Red and Voges–Proskauer reactions, ONPG hydrolysis, urease activity, and the hydrolysis of gelatin and starch. Furthermore, its capacity to utilize various carbon sources was assessed by testing for acid production from D-fructose, D-galactose, D-glucose, lactose, maltose, D-mannose, sucrose, and trehalose [26], as well as for growth on D-cellobiose, D-fructose, D-galactose, D-glucose, lactose, maltose, D-mannitol, D-mannose, sorbitol, starch, sucrose, D-trehalose, or succinate as sole carbon sources.

### 3.3. Phylogenetic Analysis

To determine the phylogenetic position of strain DWK9^T^, we conducted a dual-level analysis based on both the 16S rRNA gene and a core set of housekeeping genes. Genomic DNA was extracted from fresh biomass using a Bacterial DNA Extraction Kit (TransGen Biotech Co., Ltd., Beijing, China). The nearly complete 16S rRNA gene was amplified by PCR using the universal bacterial primers 27F (5′-AGAGTTTGATCCTGGCTCAG-3′) and 1492R (5′-TACGGYTACCTTGTTACGACTT-3′) [27] in a 20 µL reaction mixture containing Ex Taq reagent (TaKaRa Biotechnology Co., Ltd., Dalian, China) and approximately 50 ng of genomic DNA. After verification via agarose gel electrophoresis, the purified PCR product was sequenced by the Sanger method (Sangon Biotech), and the resulting sequence was deposited in GenBank (accession PV570381).

For the phylogenetic reconstruction, the 16S rRNA gene sequence of DWK9^T^ was aligned with those of closely related type species from the EzBioCloud [28] and NCBI databases using the ClustalW algorithm implemented in MEGA v11.0. Phylogenetic trees were then constructed in MEGA v11.0 (Molecular Evolutionary Genetics Analysis v 11) [29] using the neighbor-joining (NJ), maximum-likelihood (ML), and maximum-parsimony (MP) methods, with topological stability assessed by bootstrap analysis with 1000 replicates (Figures S1–S3). To provide a more robust phylogenetic framework, a core genome tree was subsequently constructed based on the concatenated sequences of 31 essential housekeeping genes (*dnaG*, *frr*, *infC*, *nusA*, *pgk*, *pyrG*, *rplA*, *rplB*, *rplC*, *rplD*, *rplE*, *rplF*, *rplK*, *rplL*, *rplM*, *rplN*, *rplP*, *rplS*, *rplT*, *rpmA*, *rpoB*, *rpsB*, *rpsC*, *rpsE*, *rpsI*, *rpsJ*, *rpsK*, *rpsM*, *rpsS*, *smpB*, and *tsf*). These sequences were retrieved from the annotated genomes of strain DWK9^T^ and its relatives, aligned, and concatenated using tools on the Majorbio Cloud Platform (Shanghai Majorbio Bio-pharm Technology Co., Ltd., Shanghai, China), with the final tree reconstructed using the NJ method in MEGA v11.0 (Figure S4).

To provide a highly robust, whole-genome-based phylogenetic framework, a phylogenomic tree was reconstructed using a set of strictly conserved single-copy marker genes. Reference genome sequences of related *Halomonas* type strains were retrieved from the NCBI RefSeq/GenBank database. The identification, alignment, and concatenation of 120 ubiquitous bacterial single-copy marker genes (bac120 set) were performed using the classify_wf workflow in GTDB-Tk v 2.7.2 (Australian Centre for Ecogenomics, The University of Queensland, Brisbane, Australia) [30]. Subsequently, the multiple sequence alignment (MSA) output was used to infer a maximum-likelihood (ML) phylogenomic tree using IQ-TREE v 2 [31]. The best-fit amino acid substitution model was automatically selected by the integrated ModelFinder in IQ-TREE. Branch support was assessed using the Ultrafast Bootstrap approximation (UFBoot) with 1000 replicates. Finally, the resulting phylogenetic tree was visualized and annotated using the interactive Tree of Life (iTOL) online platform [32].

### 3.4. Chemotaxonomy Analysis

For chemotaxonomic classification, the cellular fatty acid profile of strain DWK9^T^ was determined by gas chromatography. Biomass was harvested from cells grown on LB agar plates for 3 days at 28 °C. Approximately 50 mg of cells was then subjected to saponification, acidification, and esterification to generate fatty acid methyl esters (FAMEs) following the standard protocol of the Sherlock Microbial Identification System (MIDI, Inc., Newark, DE, USA). The resulting FAMEs were separated and identified on an Agilent 6890 gas chromatograph (Agilent Technologies, Inc., Santa Clara, CA, USA) equipped with an Ultra 2 column (25 m × 0.2 mm × 0.33 μm) and a flame ionization detector [33]. Chromatographic separation was achieved with helium as the carrier gas, using a programmed temperature gradient that started at an initial oven temperature of 170 °C and ramped at 5 °C·min^−1^ to a final temperature of 260 °C. Peak identification and quantification were performed using the MIDI Sherlock software (v6.2) against the TSBA6 database library.

### 3.5. Genome Sequencing, Assembly, and Annotation

To move beyond basic taxonomic classification and decipher the genetic blueprint underlying its robust stress tolerance and unique nitrogen metabolism, whole-genome sequencing was performed. The strain was first cultivated on LB agar for 24 h at 28 °C, after which a single colony was used to inoculate a 200 mL LB broth culture, incubated under the same conditions with shaking at 180 rpm. Cells were harvested by centrifugation (12,000× *g* for 10 min), and genomic DNA was extracted using a magnetic bead-based kit (TaKaRa Biotechnology Co., Ltd., Dalian, China). Paired-end libraries with an average insert size of ~400 bp were constructed using the NEXTFLEX Rapid DNA-Seq Kit (Bioo Scientific, Austin, TX, USA), which involved standard steps of end-repair, A-tailing, adapter ligation, and PCR amplification. The libraries were then sequenced on an Illumina NovaSeq 6000 platform (Illumina, Inc., SanDiego, CA, USA) by MajorBio to generate 2 × 150 bp paired-end reads.

All downstream bioinformatic analyses were conducted on the Majorbio Cloud Platform. The resulting raw reads were first quality-controlled and filtered using fastp (v0.23.0) [34] and subsequently assembled de novo into a draft genome using SOAPdenovo2 (v2.04) and gap-filled using GapCloser (v1.12). To ensure the robust reliability of downstream taxogenomic analyses, the quality of the assembled genome was assessed using CheckM (v1.2.2). The evaluation revealed a high completeness of 97.3% and a remarkably low contamination rate of 0.86%, confirming its high quality. This draft genome has been deposited in the NCBI database under BioProject PRJNA992788 and accession number JAVHNP000000000. A comprehensive annotation pipeline was then applied. Coding sequences (CDSs) were predicted with Glimmer (v3.02) [35], while tRNA and rRNA genes were identified with tRNAscan-SE (v2.0) and Barrnap (v0.9), respectively. Functional roles were assigned by aligning predicted protein sequences against multiple public databases (NR, Swiss-Prot, Pfam, GO, COG, KEGG, CAZY) using BLASTP (v2.3.0) and Diamond (v0.8.35). Biosynthetic gene clusters were identified using antiSMASH (v6.0) [36], and a circular genome map was generated with CGView [37] for visualization.

Finally, for definitive taxonomic placement, Average Nucleotide Identity (ANI) values were calculated against related type strains on the Majorbio Cloud Platform, which implements the OrthoANIu algorithm, a recognized standard for species delineation [17]. The digital DNA–DNA hybridization (dDDH) value was calculated using the Genome-to-Genome Distance Calculator (GGDC 3.0, DSMZ, Brauschweing, Germany).

### 3.6. Cultivation Experiment and Analysis of Extracellular Dissolved Nitrogen

Strain DWK9^T^ was cultivated in denitrifying medium (DM) to monitor bacterial growth and changes in extracellular inorganic nitrogen concentrations. Three independent biological replicate cultures were established for each treatment. The same cultures were sampled on days 1, 3, and 7. Uninoculated sterile DM was incubated in parallel as the abiotic control. Cultures were inoculated at the same initial cell density and incubated at 28 °C with shaking at 180 rpm. Based on the verified DM composition, the theoretical initial concentrations of NH_4_^+^-N and NO_3_^−^-N were 78.57 and 138.61 mg N/L, respectively.

Bacterial growth was monitored by measuring OD_600_ during the first 72 h. Extracellular NH_4_^+^-N, NO_2_^−^-N, and NO_3_^−^-N concentrations were determined on days 1, 3, and 7. At each sampling point, culture samples were centrifuged to remove bacterial cells, and the resulting cell-free supernatants were used for dissolved nitrogen analysis. NH_4_^+^-N, NO_2_^−^-N, and NO_3_^−^-N concentrations were determined using a gas-phase molecular absorption spectrometer (AJ-3700, Shanghai Anjie Environmental Technology Co., Ltd., Shanghai, China) with calibration curves prepared from standard solutions. All nitrogen concentrations are reported as mg N/L.

For each inorganic nitrogen species, the same three biological replicate cultures were sampled on days 1, 3, and 7. Data were therefore analyzed using a two-factor repeated-measures ANOVA, with treatment (inoculated culture versus sterile control) as the between-subject factor, time as the within-subject factor, and treatment × time as their interaction. For outcomes with a significant interaction, Holm-adjusted pairwise comparisons were used to test treatment differences within each sampling time. Because several NO_2_^−^-N values were close to the analytical zero, NO_2_^−^-N was analyzed after signed square-root transformation. Statistical significance was set at *p* < 0.05. Statistical analyses were performed using GraphPad Prism (v11.0.2, GraphPad Software, Boston, MA, USA).

## 4. Conclusions

Based on polyphasic taxonomic and phylogenomic evidence, strain DWK9^T^ represents a novel species within the genus *Halomonas*, for which the name *Halomonas shizuishanensis* sp. nov. is proposed. Its distinct taxonomic status is supported by its phylogenomic placement; low genomic relatedness to the closest relatives (ANI, 88.12%; dDDH, 32.50%); genomic G+C content of 55.9%; and differentiating phenotypic and chemotaxonomic characteristics, including a relatively high C16:0 proportion and negative oxidase activity. Beyond its taxonomic novelty, DWK9^T^ exhibited broad NaCl and pH growth ranges and harbored the *ectABC* cluster together with genes associated with nitrogen-related processes. During cultivation, extracellular NH_4_^+^-N concentrations were lower in inoculated cultures than in the corresponding sterile controls on days 3 and 7, whereas NO_2_^−^-N accumulated only transiently. Together, these findings establish the taxonomic identity of DWK9^T^ and provide a genomic and physiological basis for understanding its adaptation to saline–alkaline conditions and its associated extracellular nitrogen dynamics. Further studies directly resolving nitrogen fate and pathway activity, together with evaluation under defined saline–alkaline conditions, will be necessary to determine whether these characteristics can support practical applications in nitrogen management.

## 5. Patent

A Chinese patent application related to strain DWK9ᵀ and its potential applications has been filed by the authors (application No. 202410252922.5).

## Figures and Tables

**Table 1:** Differential characteristics between strain DWK9^T^ and related species of *Halomonas*.

Characteristic	1	2	3	4	5	6	7	8	9
habitat	saline–alkaline soil	lake sediment	marine sediment	saline–alkaline soil	saline–alkaline soil	marine	hypersaline lakes	salt pool	Low-temperature hydrothermal fluid
Growth properties									
Temperature for growth (°C)									
Range	4–40	2–37	4–45	4–60	4–40	4–45	4–45	5–50	2–40
Optimum	28	30–33	30	35	35	28	28	37	30
Salt concentration for growth (%, *w*/*v*)									
Range	0–20	0–20	0–20	0–15	0.2–15	0–20	0–20	0–20	0.5–22
Optimum	9	7.5	2–8	3	4	0.5–7	1–3	10	4–7
pH for growth									
Range	7.0–10.0	6.0–9.0	6.0–10.0	6–12	5.0–10.0	5.0–10.0	5.0–10.0	7.5–10.0	5.0–12.0
Optimum	8.0–9.0	7.0	7.0–9.0	9	7	7.0–7.8	7.5	9	7.5
Other phenotypic characteristics									
Oxidase	−	+	+	+	−	+	+	+	+
Catalase	+	+	+	+	+	+	+	w+	+
H_2_S production	+	+		+	+	+	+	w+	−
Nitrate reduction	+	+	−	+	+	+	−	+	+
Methyl Red	−	−	−	−	−	−	ND	ND	−
Voges–Proskauer	−	−	−	−	+	−	ND	ND	−
decarboxylase									
β-galactosidase (ONPG)	−	−	−	−	−	−	ND	ND	ND
Urea	−	−	+	−	−	+	+	−	−
Hydrolysis of									
Gelatin	−	−	−	−	+	−	−	−	−
Starch	−	−	−	−	−	−	+	−	−
Indole production	+	−	−	−	−	−	+	−	−
Aesculin	−	−	−	ND	−	−	−	ND	−
Acid production from									
D-Fructose	+	−	ND	+	−	−	+	−	−
D-Galactose	+	−	ND	−	−	−	+	−	−
D-Glucose	−	−	ND	+	−	−	+	ND	+
Lactose	−	−	ND	−	−	−	+	−	−
Maltose	−	−	ND	−	−	−	+	−	−
D-Mannose	−	−	ND	−	−	−	+	ND	−
Sucrose	−	+	ND	+	−	−	+	+	−
Trehalose	+	−	ND	+	−	−	+	+	−
Growth on									
D-Cellobiose	−	+	ND	−	−	+	−	+	−
D-Fructose	+	−	+	+	+	−	−	+	+
D-Galactose	+	−	−	−	−	+	+	+	+
D-Glucose	−	+	−	+	+	−	+	+	+
Lactose	+	−	−	ND	−	−	−	+	−
Maltose	−	−	−	+	−	−	+	+	+
D-Mannose	+	+	+	ND	+	+	−	+	+
D-Mannitol	−	+	+	w+	−	+	+	−	+
Sorbitol	+	−	−	+	−	+	−	−	−
Sucrose	−	+	+	ND	−	+	+	+	+
D-Trehalose	+	+	+	ND	−	+	+	+	+
Succinate	+	−	+	ND	+	+	+	ND	−

Strains: 1, DWK9^T^; 2, *H. lutescens *Q1UT^T^ (=KP259554^T^) [11]; 3, *H. lionensis *RHS90^T^ (=HE661586^T^) [13]; 4, *H. zhaodongensis *NEAU-ST10-25T^T^ (=JQ762286^T^) [12]; 5, *H. songnenensis *NEAU-ST10-39^T^ (=PVTK00000000) [14]; 6, *H. venusta DSM *4743^T^ (=NR042069) [15]; 7, *H. meridiana DSM *5425^T^ (=AJ306891) [15]; 8, *H. alkaliphila *18bAG^T^ (=CP024811) [16]; 9, *H. hydrothermalis Slthf2^T^* (CP023656) [15]. The symbols are defined as follows: “−”, negative; “+”, positive; “w+”, weakly positive; and “ND”, not determined.

**Table 2:** The fatty acid profiles of strain *H. shizuishanensis *DWK9^T^ compared with close relatives.

Fatty Acids	1	2	3	4	5	6	7
C10:0	-	0.6	-	2.1	3.7	2.2	0.5
C12:0	-	-	-	0.3	0.9	1.2	1
C12:0 3-OH	-	5.4	6.25	2.8	4.9	6.6	6.3
C14:0	0.3	4.2	-	2.9	4	2.6	3.3
C16:1ω7c	17.25	14.8	-	7.7	18.9	11.5	10.4
C16:0	19.64	10.1	11.85	17.6	16.3	14	14.8
C17:0 cyclo	3.26	-	4.32		-	-	0.7
C18:1ω7c	53.05	58.6	48.6	62.3	47.2	61.9	60.5
C18:0	0.58	1.2	-	0.6	-	-	-
C19:0 cyclow8c	0.3	-	9.32		0.1	-	2.2

Strains: 1, DWK9^T^; 2, *H. lutescens *Q1UT^T^ (=KP259554^T^); 3, *H. lionensis *RHS90^T^ (=HE661586^T^); 4, *H. zhaodongensis *NEAU-ST10-25T^T^ (=JQ762286^T^); 5, *H. songnenensis *NEAU-ST10-39^T^ (=PVTK00000000); 6, *H. venusta *DSM 4743^T^ (=NR042069); 7, *H. meridiana *DSM 5425^T^ (=AJ306891).

**Table 3:** Whole genomic comparison of strain DWK9^T^ with close relatives.

Reference Genome	G+C Content (%)	ANI (%)	dDDH	Total Length (Mb)
strain DWK9^T^	55.9			3.81
*H. lutescens* Q1UT^T^	61.5	88.12	32.5	3.70
*H. lionensis RHS90^T^*	54.4	86.77	/	3.65
*H. zhaodongensis *NEAU-ST10-25T^T^	53.8	83.52	20.3	3.72
*H. songnenensis *NEAU-ST10-39^T^	57.4	83.82	/	3.69
*H. venusta *DSM 4743^T^	52.3	83.36	20.9	2.76
*H. meridiana *DSM 5425^T^	59.5	85.99	/	3.86
*H. alkaliphila *18bAG^T^	53.0	83.45	20.8	4.03
*H. hydrothermalis *Slthf2^T^	56.3	84.62	/	3.84

**Table 4:** Description of *Halomonas shizuishanensis* sp. nov.

Species name	*Halomonas shizuishanensis*
Genus’s name	*Halomonas*
Specific epithet	*shizuishanensis*
Species status	sp. nov.
Species etymology	*Halomonas shizuishanensis* (shi.zhui.shan.en’sis. N.L. fem. adj. *shizuishanensis*, pertaining to Shizuishan, a city in Ningxia, China, where the type strain was isolated; D.W.K. are the initials of Dawukou, the district of isolation, and 9 denotes the strain number)
Description of the new taxon and diagnostic traits	Cells are Gram-stain-negative, aerobic, non-spore-forming, and motile rods (0.5–0.8 µm × 1.2–2.0 µm) with a single polar flagellum. On LB agar, colonies are smooth, moist, yellowish-white, and circular with entire margins. Growth occurs at 4–40 °C (optimum 28 °C), tolerates 0–20% (*w/v*) NaCl (optimum 9%), and ranges from pH 7.0–10.0 (optimum 8.0–9.0). Catalase, nitrate reduction, H_2_S production, and indole production are positive. Oxidase; ONPG; Methyl Red test; Voges–Proskauer test; and the hydrolysis of gelatin, starch, aesculin, and urea is negative. Acid is produced from D-fructose, D-galactose, and trehalose, but not from D-glucose, lactose, maltose, D-mannose, or sucrose. Can utilize D-fructose, D-galactose, lactose, D-mannose, sorbitol, D-trehalose, and succinate as sole carbon sources, but cannot utilize D-cellobiose, D-glucose, maltose, D-mannitol, or sucrose. The major cellular fatty acids are C18:1ω7c, C16:0, and C16:1ω7c. The proportion of C16:0 (19.64%) is diagnostically elevated compared to closely related species, and C17:0 cyclo is notably present. The DNA G+C content of the type strain is 55.9 mol%.
Country of origin	China
Region of origin	The saline–alkaline soil in Dawukou District, Shizuishan City, Ningxia Hui Autonomous Region, China
Date of isolation	4 October 2022
Source of isolation	Surface saline–alkaline soil
Latitude	38°53′52.638″ N
Longitude	106°19′39.222″ E
16S rRNA gene accession number	PV570381
Genome accession number	JAVHNP000000000
Genome status	Draft genome sequence
Genome size	3,810,000 bp
G+C %	55.9
Number of strains in study	1
Designation of the type strain	DWK9^T^
Strain collection numbers	CGMCC27980^T^ and KCTC 18764^T^

## Data Availability

The original contributions presented in this study are included in the article/Supplementary Materials. Further inquiries can be directed to the corresponding authors.

## Supplementary Materials

The following supporting information can be downloaded at https://www.mdpi.com/article/10.3390/ijms27188335/s1.
