# Supplementary material for: Taxogenomic and Physiological Characterization of Halomonas shizuishanensis sp. nov. Reveals Osmotic-Adaptation Traits and an Ammonium-Utilization Phenotype
Source: Int J Mol Sci. 2026 Sep 19;27(18):8335. doi: 10.3390/ijms27188335 (PMC13607790; doi:10.3390/ijms27188335)
Supplement: Supplementary file 1 [file ijms-27-08335-s001.zip › ijms-4374599-supplementary.pdf]

Supplementary Material for

Taxogenomic and Physiological Characterization of *Halomonas shizuishanensis* sp. nov. Reveals Osmotic-Adaptation Traits and an Ammonium-Utilization Phenotype

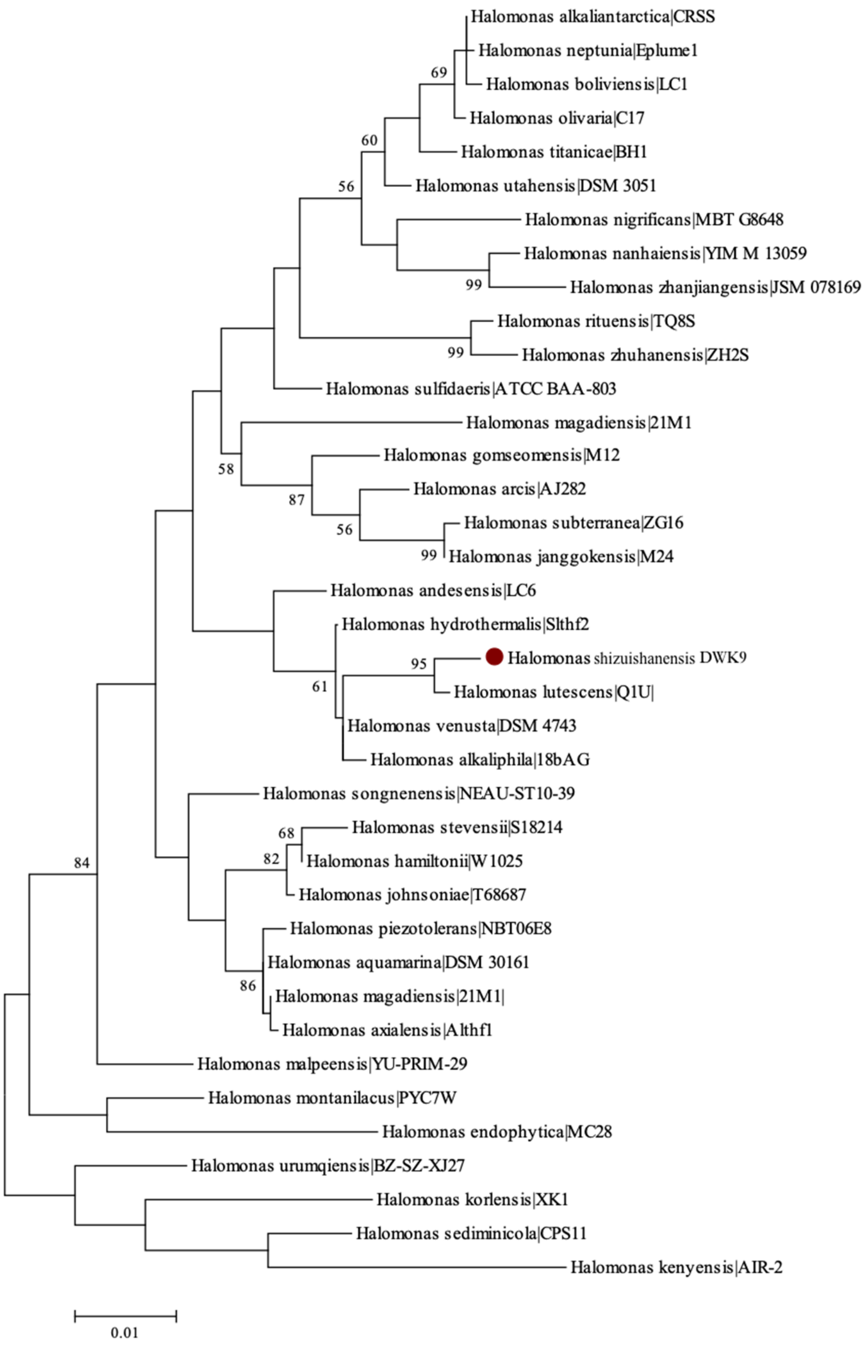

Supplement Figure S1. Neighbour-joining phylogenetic tree based on 16S rRNA gene sequences showing the relationships between strain DWK9T and members of the genus Halomonas. Bootstrap percentages (≥70 %) based on 1000 re-samplings are listed at the nodes. The 16S rRNA gene sequence of strain AIR-2 was used as an outgroup.

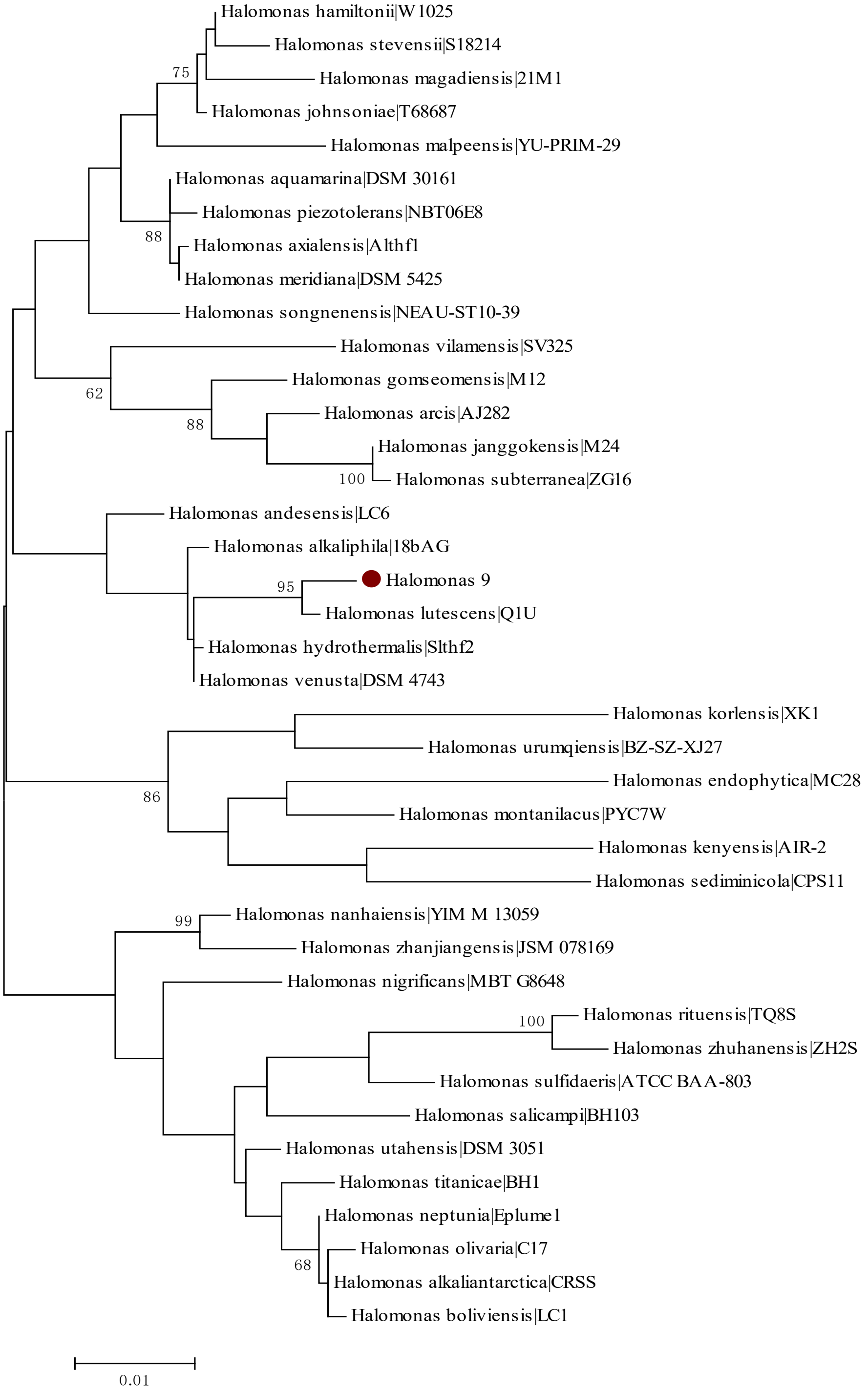

Supplement Figure S2. The maximum-likelihood phylogenetic trees.

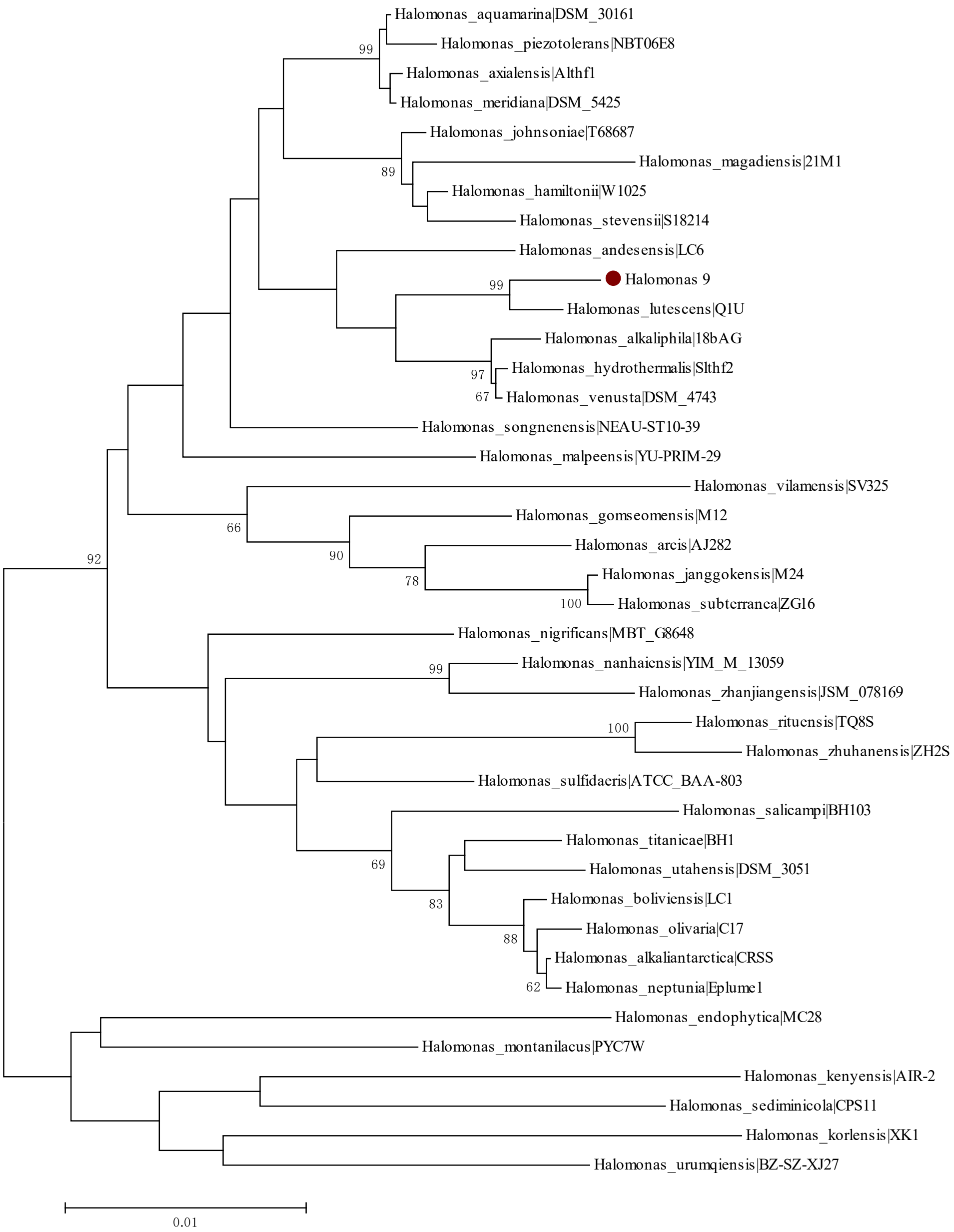

Supplement Figure S3. The maximum- parsimony phylogenetic trees.

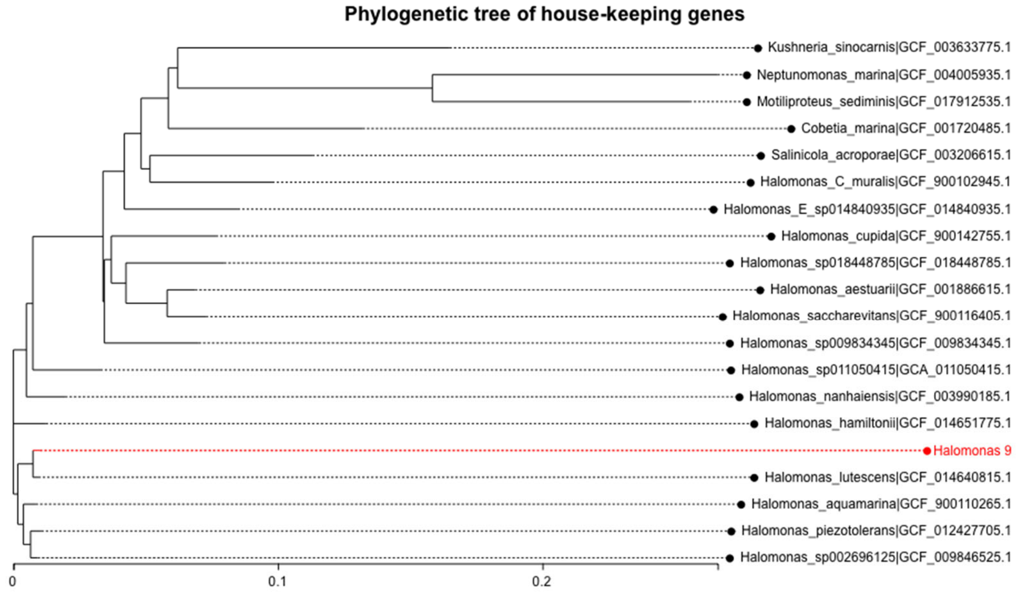

Supplement Figure S4. Neighbour-joining phylogenetic tree based on house-keeping gene sequences showing the relationships between strain DWK9^{T} and members of the genus Halomonas. The house-keeping gene contains dnaG, frr, infC, nusA, pgk, pyrG, rplA, rplB,rplC, rplD, rplE, rplF, rplK, rplL, rplM, rplN, rplP, rplS, rplT, rpmA, rpoB, rpsB, rpsC,rpsE, rpsI, rpsJ, rpsK, rpsM, rpsS, smpB, tsf.

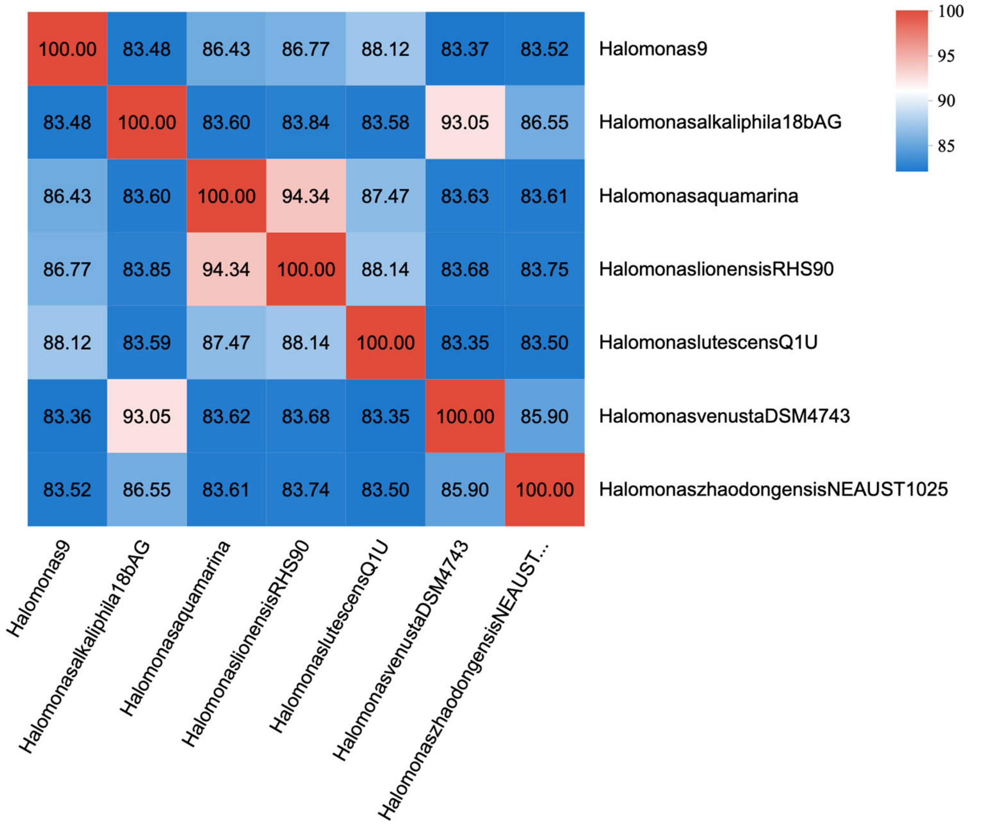

Supplement Figure S5. Species identification for the genome of *Halomonas shizuishanensis DWK9^T*. The heatmap shows the color-coded ANI values between each pair of genomes from the nine species within the genus *Halomonas*. For each species, genomes retrieved from NCBI Assembly database were used for the comparative analysis.

Supplement Table S1. Genomic features of *Halomonas shizuishanensis DWK9^T*.

Species	Genome size	Total Scaf No.	Functional genes	rRNAs	tRNAs
DWK9^T	3.81 Mb	12	3,468	5	56

Supplement Table S2. Gene annotation overview of *Halomonas shizuishanensis DWK9^T* using different databases.

Name	NR	Swiss-Prot	Pfam	COG	GO	KEGG
Halomonas shizuishanensis DWK9^T	3439	2780	3114	3025	1896	2303
